# A Retrospective Whole-Genome Sequencing Analysis of Carbapenem and Colistin-Resistant *Klebsiella pneumoniae* Nosocomial Strains Isolated during an MDR Surveillance Program

**DOI:** 10.3390/antibiotics9050246

**Published:** 2020-05-12

**Authors:** Bernardina Gentile, Antonella Grottola, Gabriella Orlando, Giulia Fregni Serpini, Claudia Venturelli, Marianna Meschiari, Anna Anselmo, Silvia Fillo, Antonella Fortunato, Florigio Lista, Monica Pecorari, Cristina Mussini

**Affiliations:** 1 Scientific Department, Army Medical Center, Via Santo Stefano Rotondo 4, 00184 Rome, Italy; dinagentile629@gmail.com (B.G.); annanselm@gmail.com (A.A.); silviafillo@gmail.com (S.F.); antonellafortunato75@gmail.com (A.F.); romano.lista@gmail.com (F.L.); 2 Molecular Microbiology and Virology Unit, University Hospital Policlinico Modena, Via Del Pozzo, 71, 41124 Modena, Italy; grottola.antonella@policlinico.mo.it (A.G.); fregniserpini.giulia@policlinico.mo.it (G.F.S.); pecorari.monica@policlinico.mo.it (M.P.); 3 Department of Surgery, Medicine, Dentistry, and Morphological Sciences with Transplant Surgery, Oncology, and Regenerative Medicine Relevance, University of Modena and Reggio Emilia, Via Del Pozzo 71, 41124 Modena, Italy; cristina.mussini@unimore.it; 4 Infectious Disease Clinic, University Hospital Policlinico Modena, Via Del Pozzo 71, 41124 Modena, Italy; mariannameschiari1209@gmail.com; 5 Clinical Microbiology Unit, University Hospital Policlinico Modena, Via Del Pozzo, 71, 41124 Modena, Italy; venturelli.claudia@policlinico.mo.it

**Keywords:** *Klebsiella pneumoniae*, MDR nosocomial spread, whole-genome sequencing, genetic relatedness

## Abstract

Multidrug-resistant *Klebsiella pneumoniae* (MDR *Kp*), in particular carbapenem-resistant *Kp* (CR-*Kp*), has become endemic in Italy, where alarming data have been reported on the spread of colistin-resistant CR-*Kp* (CRCR-*Kp*). During the period 2013–2014, 27 CRCR-*Kp* nosocomial strains were isolated within the Modena University Hospital Policlinico (MUHP) multidrug resistance surveillance program. We retrospectively investigated these isolates by whole-genome sequencing (WGS) analysis of the resistome, virulome, plasmid content, and core single nucleotide polymorphisms (cSNPs) in order to gain insights into their molecular epidemiology. The in silico WGS analysis of the resistome revealed the presence of genes, such as *blaKPC*, related to the phenotypically detected resistances to carbapenems. Concerning colistin resistance, the plasmidic genes *mcr* *1–9* were not detected, while known and new genetic variations in *mgrB*, *phoQ*, and *pmrB* were found. The virulome profile revealed the presence of type-3 fimbriae, capsular polysaccharide, and iron acquisition system genes. The detected plasmid replicons were classified as *IncFIB(pQil)*, *IncFIB(K)*, *ColRNAI*, *IncX3*, and *IncFII(K)* types. The cSNPs genotyping was consistent with the multi locus sequence typing (MLST) and with the distribution of mutations related to colistin resistance genes. In a nosocomial drug resistance surveillance program, WGS proved to be a useful tool for elucidating the spread dynamics of CRCR-*Kp* nosocomial strains and could help to limit their diffusion.

## 1. Introduction

*Klebsiella pneumoniae* (*Kp*) is a Gram-negative bacterium that can colonize or cause infections in hospitalized patients. Multidrug-resistant (MDR) *Kp* strains show high-level resistance to β-lactams, aminoglycosides, quinolones, tigecycline, and colistin. In particular, the carbapenem-resistant *Kp* (CR-*Kp*) pathogen represents a worldwide challenge due to its high mortality rates. It has become endemic in Italy, where there have been several reports of hospital outbreaks [1,2,3,4,5].

Various carbapenem resistance mechanisms have been identified; however, the mechanism that most frequently occurs is related to *Kp* carbapenemase (KPC) production [6].

The increasing spread of nosocomial MDR *Kp* has led to the reintroduction of colistin, which is one of the few widely available therapeutic options for CR-*Kp* infections [7]. As a consequence of this renewed use, the isolation of colistin-resistant CR-*Kp* (CRCR-*Kp*) strains has gradually increased in Italy [8,9].

Prior to 2015, colistin resistance had only been linked to mutational and regulatory changes mediated by chromosomal genes [10]. Gene modifications involved in efflux pump component encoding have also been correlated with colistin resistance [11]. Moreover, plasmid-encoded colistin resistance genes have been reported to be transmissible resistance mechanisms in *Enterobacteriaceae.* The presence of colistin resistance genes in mobile genetic elements poses a significant public health risk, as these genes can spread rapidly by horizontal transfer and require global monitoring and surveillance [12].

Methods for discriminating and characterizing different *Kp* isolates are essential to the optimization of infection control resources. Systems based on phenotypes (serotype, biotype, or antibiogram) and molecular methods (multi locus sequence typing (MLST), pulsed-field gel electrophoresis (PFGE), and repetitive extragenic palindromic PCR (rep-PCR)) have been used for many years. Despite the increase in discrimination power from MLST to methods that interrogate the entire genome, such as PFGE and rep-PCR, these techniques may not provide sufficient resolution between strains due to *Kp*’s high clonality [13].

This limitation has been overcome with improvements in sequencing technologies. Whole-genome sequencing (WGS) is positioned to become an essential epidemiological and clinical tool for day-to-day infection control and, for some pathogens, a method for detecting the molecular mechanisms that underlie antibiotic resistance, virulence factors, and plasmid diffusion [14].

At Modena University Hospital Policlinico (MUHP), a 677-bed tertiary care hospital in northern Italy, CR-*Kp* has been continuously isolated since 2008, and a joint infection control and antimicrobial stewardship program was initiated in 2012. During the period 2013–2014, 27 CRCR-*Kp* nosocomial strains were isolated and investigated by MLST as part of the surveillance and infection control program.

The objective of this study was to use in silico WGS to retrospectively analyze these strains in order to better understand CRCR-*Kp* spread dynamics. We used web tools and bioinformatics software to characterize the resistome, virulome, plasmid content, and core single nucleotide polymorphisms (cSNPs). WGS data were then correlated to the clinical–epidemiologic context.

## 2. Results

### 2.1. Antimicrobial Susceptibility, Carbapenemase Phenotype Detection and MLST

All isolates showed resistance to carbapenems, beta-lactams, ciprofloxacin, fosfomycin, aminoglicosyde, and colistin. Additionally, 21 of 27 (78%) and 18 of 27 (67%) strains were found to be resistant to trimethoprim-sulphonamide and to tigecycline, respectively. Phenotype testing for carbapenemase showed that all isolates were class A carbapenemases (Table 1 and Supplementary Table S1).

The MLST showed that all samples belonged to Clonal Complex (CC) 258. Twenty-five strains were assigned to sequence type (ST) 512, and two strains were assigned to ST258 (Table 1).

### 2.2. Whole-Genome Sequencing and in Silico Data Analysis

The assembly statistics for each genome are reported in Supplementary Table S2.

#### 2.2.1. Resistome Analysis

**Figure 1:**
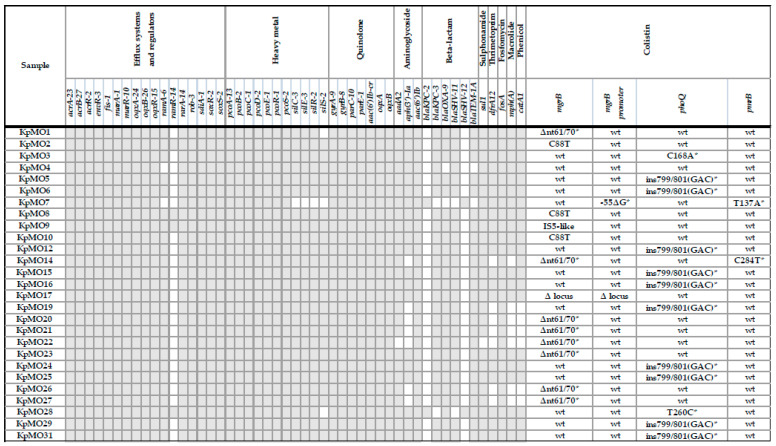
Resistome data. Resistome data obtained by ResFinder-2.1 software, Pasteur MLST *Kp* database and running BLAST are here summarized. In particular, in the left box, grey and white colors represent gene presence and absence respectively; in the right box the colistin-resistance related mutations are grouped.

The data on antibiotic resistance due to the presence/absence of genes and the mutations that were detected in colistin resistance chromosomal determinants are reported in Figure 1.

The revealed genes are grouped with respect to the related resistance mechanism. Concernig colistin resistance, many different mechanisms have been reported. Prior to 2015, colistin resistance had only been linked to mutational and regulatory changes mediated by chromosomal genes controlling lipopolysaccharide (LPS) modifications. In *Kp*, the LPS modification is mediated by the activation of different two-component regulatory systems (TCRSs): PmrA/PmrB, PhoP/PhoQ, and CrrA/CrrB. TCRS mutations can cause constitutive expression of the *pmrCAB* and *pmrHFIJKLM* operons. Moreover, inactivation of the PhoQ/PhoP negative regulator encoded by *mgrB* has been suggested to play a prominent role in colistin-resistance in *Kp* [10] Moreover, plasmid-encoded *mcr1–mcr9* genes have been reported as a transmissible resistance mechanism in *Enterobacteriaceae* [12]. 

The *mcr* genes absence and the neutral mutations are not presented in the figure. * New mutations in colistin-resistance related genes detected in this study.

The percentage similarity in the alignment between the best-matching resistance gene in ResFinder and the corresponding sequence in the input genome ranged from 97.14 to 100, with a 100% query/high-scoring segment pair (HSP) length.

We found the presence of genes associated with resistance to aminoglycosides (*aadA2, aph(3’)-Ia*, and *aac(6′)-Ib*), quinolones (*oqxA, oqxB, aac(6′)-Ib-cr, gyrA-B*, and *parC-E*), macrolide (*mphA*), sulfhonamide (*sul1*), fosfomycin (*fosA*), and trimethoprim (*dfrA-12*). Alleles of multidrug efflux system and regulator genes (*acrA-B-R, envR, fis, marA-R, oqxA-B-R, ramA-R, rarA, rob, sdiA,* and *soxR-S*) and heavy metal resistance determinants (*pcoA-B-C-D-E-R-S* and *silC-E-R-S*) were quite uniformly detected as reported in Figure 1.

The β-lactamase characterization demonstrated the presence of *blaKPC-2/blaSHV-12* and *blaKPC-3/blaSHV-11* in the ST258 and ST512 strains, respectively. *blaTEM-1A* and *blaOXA-9* were present in all but two isolates (KpMO4 and KpMO7).

Concerning genetic determinants related to colistin resistance, the BLAST results and ResFinder analysis revealed several genetic modifications in chromosomal *loci* and the absence of the plasmidic genes *mcr 1–9*, respectively. Compared with the *Kp*-ST512-K30BO and *Kp*-HS11286 reference sequences, all isolates showed wild-type *acrAB, pmrHFIJKLM, crrA, kpnEF, lpxM, phoP,* and *pmrACD loci.* All samples showed two neutral *crrB* point mutations (the silent A84C and the missense A887T, corresponding to the neutral amino acid change L296Q in the CrrB protein) and two silent *pmrD* point mutations (T162C and C195T) with respect to the ST11-HS11286 reference sequence.

As shown in Figure 1, significant new alterations were found in *mgrB*, *phoQ*, and *pmrB*. In particular, we found seven new mutations, including a 10-nucleotide deletion in *mgrB*, a point mutation in the *mgrB* promoter, an insertion in *phoQ*, and two point mutations in both *phoQ* and *pmrB*.

These new variants were defined by a literature search and a BLAST search, and were found to not match the *Kp* gene sequences/genomes in the GenBank database. Three different types of non-silent *mgrB* alterations (deletions, nonsense mutations, and an insertional inactivation) were detected in 13 of 27 isolates. Eight isolates (KpMO1, KpMO14, KpMO20-23, KpMO26, and KpMO27) exhibited a new 10-nucleotide (nt) deletion (Δnt61/70). This deletion causes a frame shift with consequent double amino acid (aa) substitutions (T21L and Q22T) and the production of a 22-aa, C-terminal truncated, and most likely nonfunctional MgrB protein. At the level of the *mgrB* promoter, a new point deletion, −55ΔG, was detected in KpMO7.

Sequence analysis of the *phoQ* gene revealed mutations in 12 of 27 isolates. Ten of these (KpMO5, KpMO6, KpMO12, KpMO15, KpMO16, KpMO19, KpMO24, KpMO25, KpMO29, and KpMO31) showed a new 3-nt insertion (ins799/801(GAC)) generating the addition of an aspartic acid (D266_267insD). KpMO12 also had the *phoQ* neutral mutation C1369G (the Q457E aa change). KpMO3 and KpMO28 each had one new missense mutation in *phoQ* (C168A (S56R) and T260C (L87P), respectively).

The *pmrB* gene was found to have two new missense mutations (T137A (V46E) in KpMO7 and C284T (P95L) in KpMO14). In KpMO4, no mutation that could explain the colistin resistance was found.

PROVEAN analysis predicted a deleterious impact on the biological protein function of all of the new mutations except one (C1369G in the *phoQ* gene) (Table 2).

Apart from the new mutations in colistin-related genes, we found some well-known *mgrB* resistance mechanisms, namely the C88T point mutation, a complete lack of the gene, and an insertional inactivation.

Three samples (KpMO2, KpMO8, and KpMO10) showed the previously known C88T mutation, which generates a premature stop codon and produces a truncated, nonfunctional, and 29-aa-long protein. Moreover, KpMO17 exhibited a 1879-nt *mgrB* genetic environment deletion, including a large region upstream (part of the gene encoding the major facilitator superfamily protein and the *kdgR* and *yobH* genes) and within the *mgrB* gene (the *mgrB* promoter and the first *mgrB* 132/144 coding nt, the Δ locus).

Finally, in KpMO9, *mgrB* was found to be completely disrupted by a 1196-nt insertion sequence (IS5-like) at the level of the 74–75 nt positions (best match with the AO-1367 *Kp* strain, accession No. KP967591.1) [15].

#### 2.2.2. Virulome Analysis

**Figure 2:**
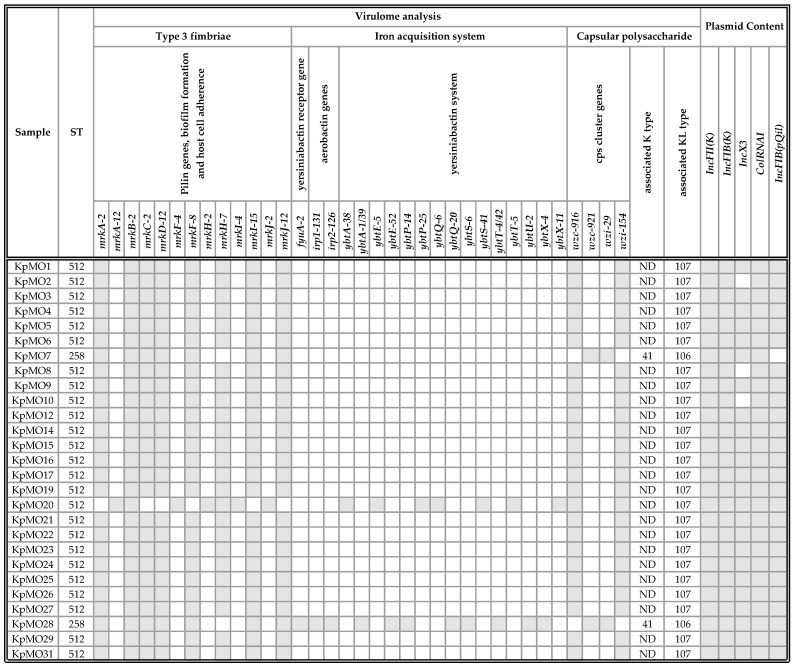
Virulome data and plasmid content. Virulome data were obtained by Pasteur MLST *Kp* database; plasmid content data were obtained by PlasmidFinder-1.3. Grey and white colors represent gene presence and absence respectively. ND, Not Defined.

The virulence repertoire was represented by type-3 fimbriae (the *mrk* operon), capsular polysaccharides (*cps* cluster genes associated with the K type (K) and the K *locus* (KL)), and iron acquisition systems (the *fyu*, *irp*, and *ybt* genes) (Figure 2).

The complete *mrk* operon was detected in all but three samples (KpMO20, *mrkCD*-defective; KpMO7 and KpMO28, *mrkH*-defective).

The ST258 (KpMO7/KpMO28) isolates had the 29/921 *wzi*/*wzc* alleles in association with K41 and KL106. All ST512 isolates had the 154/916 *wzi*/*wzc* alleles associated with not defined (ND) K and KL107. We did not find capsule synthesis *loci* associated with hypervirulent serotypes (i.e., serotypes K1, K2, and K5).

Iron acquisition genes were found in two samples only. A complete yersiniabactin siderophore system (*fyuA*, *irp1*, *irp2*, and the *ybt* operon) was found in KpMO28, while an incomplete *ybt* cluster (Δ*ybtU*) was detected in KpMO20.

#### 2.2.3. Plasmid Content Analysis

Five replicon types were globally detected and characterized as *IncFII(K)*, *IncFIB(K)*, *ColRNAI*, *IncX3*, and *IncFIB(pQil)*. The percentage similarity in the alignment between the best-matching plasmid in the PlasmidFinder database and the corresponding sequence in the input genome ranged from 97.97 to 100. *IncFII(K)*, *IncFIB(K)*, and *ColRNAI* replicons were identified in all isolates, while *IncFIB(pQil)* was not found in KpMO7. The absence of *IncX3* was common to KpMO2, KpMO8, and KpMO10 (Figure 2).

#### 2.2.4. Phylogenetic Analysis

We drew a maximum likelihood cSNPs tree of the 27 samples in order to provide high-resolution strain tracking and discrimination. In total, we identified 1731 SNPs, of which 1002 were shared by all samples. 

**Figure 3:**
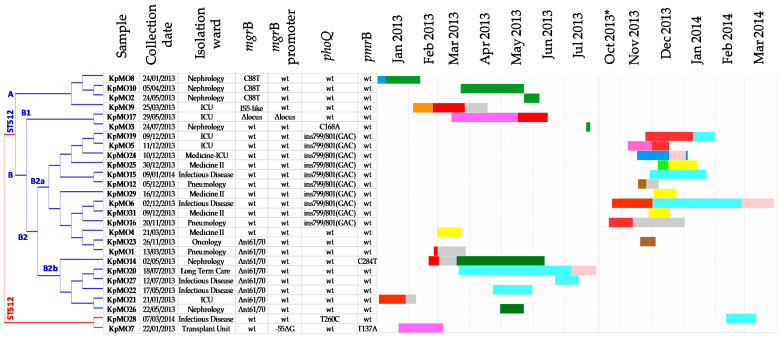
Core SNPs analysis data incorporated into the epidemiological metadata. Core SNPs tree (left) shows two major lineages corresponding to the ST512 (blue) and the ST258 (red). Clusters within ST512 lineage are indicated by capital letters (A, B) followed by a number in the minor branches (B1, B2) and by additional lowercase letters in the subgroups (B2a, B2b). The right box represents patients’ movements inside the hospital, each color represents a ward. ICU, red; Infectious Disease, cyan, Long Term Care, pink; Medicine II, yellow; Medicine ICU, blue; Nephrology, dark green; Oncology, brown; Orthopedics, light green; Otolaryngology, orange; Pneumology, grey; Transplant Unit, fuchsia. ICU, Intensive Care Unit; Δ, deletion; IS, Insertion Sequence; ins, insertion; wt, wild type. * days 15–31.

The cSNPs analysis resulted in two major lineages corresponding to the ST512 and the ST258 samples. The ST512 lineage included two major clusters, A (*n* = 4) and B (*n* = 21). The B cluster was grouped into two minor branches, B1 (*n* = 2) and B2 (*n* = 19), with the latter consisting of two subgroups, B2a (n = 13) and B2b (*n* = 6) (Figure 3).

### 2.3. The Molecular Data in the Clinical–Epidemiologic Context

The phylogenetic data, MLST results, and mutations detected in colistin resistance genes were incorporated into the epidemiological metadata in order to elucidate the routes of transmission (Figure 3).

The ST512/cluster A isolates were closely related and shared the same colistin resistance mechanism and plasmid content. The ST512/cluster B isolates were characterized by a high level of molecular and epidemiological heterogeneity, although many of them shared the same colistin-resistance-related mutation. The two ST258 isolates were unrelated to all of the other strains, and each strain had a unique colistin-resistance-related mutation.

## 3. Discussion

Our retrospective investigation of 27 CRCR-*Kp,* isolated at Modena University Hospital Policlinico, by WGS analysis of the resistome, virulome, plasmid content, and cSNPs allowed us to obtain two main results: the identification of new genetic variations in the colistin-resistance related genes and an informative epidemiological picture of the spread of CRCR-*Kp* in our clinical setting.

The MLST analysis showed that all isolates belonged to the pandemic CC258. These data confirmed the pandemic CC258′s global distribution and its ability to cause hospital outbreaks and disseminate carbapenemase genes [1,6,16]. Nevertheless, the MLST results did not allow us to identify specific relationships or possible patterns of transmission among the studied strains. On the other hand, the WGS data provided us with an adequate amount of information about the evolution of CRCR-*Kp* circulation in our hospital.

Concerning WGS resistome analysis, in all samples, genes coding for the main classes of antibiotic resistance were found to be in agreement with the susceptibility test.

In particular, and as previously reported in Italy, we found that the carbapenemase *blaKPC-3* allele-associated ST512 was more prevalent than the *blaKPC-2*-carrying ST258 [2,3,17].

Regarding genetic determinants related to colistin resistance, we confirmed, as reported by other authors, the absence of the plasmidic genes *mcr 1–9* [18]. Instead, both known and significant new genetic modifications were identified in chromosomal *loci*, specifically in the *mgrB*, *phoQ*, and *pmrB* genes. We identified some well-known chromosomal colistin resistance mechanisms involving *mgrB*, such as a C88T point mutation [10], a complete lack of the gene [10,18,19], and an insertional inactivation [10,15,19,20].

For all new mutations except one (C1369G in the *phoQ* gene), the PROVEAN analysis predicted a deleterious impact on the biological protein function, suggesting a possible association between each of these mutations and colistin resistance. This in silico analysis allowed us to overcome one limitation of our study, namely the absence of the trans-complementation tests and evaluation of the *mgrB*, *pmrB*, and *phoQ* expression levels that are commonly used to confirm an association between a new mutation and colistin resistance.

The results of the virulence analysis of the studied samples showed the presence of type-3 fimbriae known to be involved in biofilm formation on biotic and abiotic surfaces of medical devices in a hospital environment [21]. The absence of capsule synthesis *loci* associated with hypervirulent serotypes (i.e., serotypes K1, K2, and K5) indicates that MDR strains do not currently overlap with hypervirulent CCs [22]. Moreover, we confirmed that KL107 and KL106 were associated with wzi/wzc154/916 and wzi/wzc29/921, respectively [23].

Plasmids, which are considered to be the primary source of *Kp* gene variability, have been used as molecular markers in epidemiological investigations [24]. However, the homogeneity of our results limits the use of plasmid content as an epidemiological marker, except for KpMO2, KpMO8, and KpMO10, which were found to be defective for *IncX3*, and KpMO7, which was found to be defective for *IncFIB(pQil)*.

Moreover, the restrictions intrinsic to short-read technologies (e.g., Illumina) in the WGS approach did not allow us to accurately reconstruct the genomic context surrounding the repeated sequences in the plasmids [25]. Nevertheless, the variability in plasmid content found in our collection may provide support to the hypothesis that plasmidic exchanges and arrangements can occur in endemic healthcare settings, generating additional plasmid types.

The incorporation of MLST and WGS data into the clinical–epidemiologic context allowed us to draw some inferences. As CRCR-*Kp* can spread via person-to-person contact or environmental sources, the WGS analysis allowed us to identify possible transmission patterns.

For example, the ST512/cluster A isolates, which were found to be closely related, could have derived from a common source that was confined to the nephrology ward. Indeed, the KpMO8 strain, which was the first to be isolated from the nephrology ward (January 2013), was found to belong to ST512/cluster A and carry the C88T mutation in the *mgrB* gene. In the following months, the ST512/cluster A isolates evolved in the same ward as KpMO10 and KpMO2, which were found to be closely related to each other, and shared with KpMO8 the same colistin resistance mechanism and plasmid content. This result was confirmed by the characterization of plasmid content with a unique profile in which *IncX3* was absent. The KpMO9 isolate, which first appeared in the intensive care unit (ICU), slightly diverged from the other ST512/cluster A isolates, showing an *mgrB* insertional inactivation. Moreover, it had the same plasmid content as all other isolates except for KpMO7. This latter isolate was the only one found to be defective for *IncFIB(pQil).* Therefore, WGS allowed us to differentiate KpMO9 from KpMO8, KpMO10, and KpMO2, while MLST grouped them together.

The ST512/cluster B isolates were characterized by a high level of molecular and epidemiological heterogeneity.

KpMO17 and KpMO3 were isolated from different wards, and although both belonged to B1, they did not share the same variation in colistin resistance determinants.

Ten strains, isolated in five wards during the period November 2013 to January 2014, were characterized by a *phoQ* 799/801(GAC) insertion and belonged to the B2a subgroup. Eight strains, isolated from six wards, carried the *mgrB-*Δnt61/70 deletion and were isolated over a long period of time (January 2013 to November 2013). Six of these isolates belong to the B2b subgroup, while two isolates (KpMO1 and KpMO23) belong to the B2a subgroup. In the larger ST512/cluster B, the isolates’ molecular heterogeneity did not allow us to confirm the hypothesis of transmission or of exposure to the same hospital source of CRCR-*Kp*. In particular, the closely related KpMO19/KpMO5 strains were isolated from the ICU at the same time, the KpMO25/KpMO29/KpMO31 strains were isolated from Medicine II, and the KpMO20/KpMO27/KpMO22 strains were isolated from the infectious disease ward.

## 4. Materials and Methods

### 4.1. Study Design

From January 2013 to March 2014, 85 non-duplicated CRCR-*Kp* strains were isolated from several wards within the MDR surveillance program by means of universal patient rectal swab screening at admission (t = 0) and thereafter at regular intervals (weekly) during hospitalization. The isolates were immediately subjected to both routine phenotypic and traditional genotyping analysis (MLST). Of these 85 strains, 27 strains that were isolated from inpatient rectal screening swabs or clinical samples obtained during hospitalization (starting 48–72 h after admission) were selected and used for a retrospective WGS analysis in order to gain insights into the molecular epidemiology of nosocomial CRCR-*Kp* strains.

For each patient, we selected the first obtainable CRCR-*Kp* isolate, favoring more relevant clinical samples (urine, blood, etc.) where available. Table 3 contains information on the isolates and each patient’s characteristics; each isolate number corresponds to a particular case.

Nine (33.3%) patients died during their hospital stay. Five (55.5%) deaths were due to sepsis. There were only two documented cases of CRCR-*Kp* infection: one blood stream infection and one urinary tract infection.

This study was approved by Modena’s provincial ethics committee and registered with protocol no. 2655, 21/July/2016. No written informed consent was obtained from patients as all data were analyzed anonymously after a de-identification process.

### 4.2. Antimicrobial Susceptibility, Carbapenemase Phenotype Detection and MLST

At the time of strain isolation (2013–2014), species identification and antimicrobial susceptibility were determined using the Vitek2 automated system (BioMérieux, Marcy l’Etoile, France). The susceptibility test results and minimum inhibitory concentration (MIC) were interpreted according to the 2012 EUCAST breakpoints criteria [26]. Susceptibility to colistin and susceptibility to tigecycline were verified using the E-test (BioMérieux). Carbapenemase phenotype testing was performed using the KPC+MBL Confirm ID kit (Rosco Diagnostica A/S, Taastrup, Denmark).

To assign sequence types, an MLST analysis was performed using the Pasteur database scheme available for *K. pneumoniae* (http://bigsdb.pasteur.fr/klebsiella/klebsiella.html).

### 4.3. Whole-Genome Sequencing and in Silico Data Analysis

Genomic DNA for molecular analysis was extracted from the 27 CRCR-*Kp* isolates using the Maxwell-16 automated DNA/RNA extraction system (Promega, Madison, WI, USA). DNA quality, quantity, and purity were determined using agarose gel, a NanoDrop 8000 spectrophotometer (Thermo Fisher Scientific, Wilmington, DE, USA), and an E6150 Quantus™ Fluorometer (Promega).

The samples were fully sequenced by using a next-generation sequencing (NGS) approach on the MiSeq platform (Illumina, San Diego, CA, USA). The Nextera XT DNA protocol was applied with 1–1.5 ng of starting DNA, and sequencing was performed with a v3 kit (600 cycles). Paired-end reads were demultiplexed into separate samples, quality checked by removing adapter sequences and bases (quality score <25) using the FastQC (Babraham Research institute, Cambridge, UK) and Sickle software (https://github.com/najoshi/sickle), and de novo assembled into contigs using the Abyss-pe v1.5.2 program (Canada’s Michael Smith Genome Sciences Centre, Vancouver, Canada) (k parameter = 63) or the SPAdes v3.7.0 program (Center for Algorithmic Biotechnology St. Petersburg State University, St. Petersburg, Russia) [27,28]. Contigs longer than 500 bp were selected using an ad hoc script and kept for further analysis.

The 27 nucleotide sequences were deposited at DDBJ/ENA/GenBank under Bioproject ID PRJNA504600.

#### 4.3.1. Resistome, Virulome, and Plasmid Content Analysis

The resistome of all sequenced isolates was analyzed using the ResFinder-3.2 software (Center for Genomic Epidemiology, Lyngby, Denmark) (http://www.genomicepidemiology.org; identity threshold (ID) 90%) and the Pasteur MLST *Kp* database (Pasteur Institut, Paris, France). Furthermore, in order to study molecular chromosomal mechanisms of colistin resistance, *acrAB*, *pmrHFIJKLM, crrAB*, *KpnEF, lpxM, mgrB*/*mgrB* promoter, *phoPQ, pmrCAB*, and *pmrD* were analyzed by running the BLAST (National Center for Biotechnology Information, Bethesda, MD, USA) program and using as references the sequences reported in Table 4. The PROVEAN tool (J. Craig Venter Institute, La Jolla, CA, USA) (http://provean.jcvi.org) was used to predict the (neutral or deleterious) biological impact of aa substitutions/indels on protein function [29].

The virulome was investigated using the Pasteur MLST *Kp* database (https://bigsdb.pasteur.fr/cgibin/bigsdb/bigsdb.pl?db=pubmlst_klebsiella_seqdef&page=sequenceQuery).

The PlasmidFinder-1.3 web tool (Center for Genomic Epidemiology, Lyngby, Denmark) (http://www.genomicepidemiology.org; ID 95%) was used to define the replicon plasmid content type.

#### 4.3.2. Phylogenetic Analysis

To establish genetic relatedness among the 27 CRCR-*Kp* isolates, SNP discovery was performed using the kSNP v3.0 program (Bellingham Research Institute. Bellingham, WA, USA (k-mer = 21). SNP loci were defined by an oligo of k length surrounding a central SNP allele [30]. The maximum likelihood tree based on the cSNPs detected in all genomes was visualized with the Dendroscope v3.2.10 software (Center for Bioinformatics, Tübingen, Germany) [31].

### 4.4. The Molecular Data in the Clinical–Epidemiologic Context

The results obtained from both the MLST and WGS-based analyses were incorporated into the clinical–epidemiological metadata that were collected from the patients’ medical records.

## 5. Conclusions

Due to the growing importance of MDR *Kp*, a fast and accurate identification and typing of pathogens is essential for effective surveillance and outbreak detection. We need to know the genetic arrangement related to the antibiotic resistance, to understand population structure in hospital settings, and their relationship.

The most important result of this retrospective WGS analysis was the discovery of new genetic variations involving the *mgrB*, *phoQ,* and *pmrB* genes related to colistin resistance and the absence of the plasmidic *mcr* gene.

Moreover, this study proved to be useful to a program for monitoring the spread of nosocomial CRCR-*Kp* strains, and allowed us to confirm what has already been shown in many other studies [32,33,34,35,36]. In particular, the distribution of mutations in colistin resistance determinants was consistent with the cSNPs clustering. Thus, it may serve as a good epidemiological marker. As WGS was more discriminating than MLST, it allowed us to identify possible CRCR-*Kp* transmission patterns and to obtain a clustering reconstruction that was more consistent with the epidemiological analysis.

WGS produces results with an excellent cost/benefit ratio and a mean measured turnaround time (TAT) of 4.4 days comparable to TAT required for investigations with less discriminatory methodologies [33]. However, WGS-informed outbreak tracking is still usually performed only retrospectively [37].

In our view, the data obtained retrospectively in this study, if available in real-time, could have helped to surveil the alert nosocomial pathogens by directing the infection control team to focus its attention and resources on those wards where WGS would have highlighted transmission events.

Furthermore, the future routine clinical implementation in our hospital of real-time WGS would provide the infection control team with reliable and timely data about the emergence and spread of antimicrobial resistance, and hopefully the ability to prevent outbreaks by the rapid application of infection control procedures and the implementation of a targeted antimicrobial stewardship program.

Finally, even if our findings have allowed us to better understand the spread of *Kp* in hospitals at a local level, they add to the molecular CRCR-*Kp* epidemiology data at both the national and international levels and contribute to defining a framework for the epidemiology of this pathogen.

## Figures and Tables

**Table 1:** Antimicrobial susceptibility profiles, carbapenemase class and MLST of 27 CRCR-*Kp* isolates. Antimicrobial susceptibility profiles: dark grey, grey, and white colors represent resistant, intermediate and sensitive strains respectively. GEN, Gentamicin; AMK, Amikacin; IPM, Imipenem; MEM, Meropenem; ETP, Ertapenem; CIP, Ciprofloxacin; TMP/SMX, Trimethoprim/Sulphonamide; TGC, Tigecycline; COL, Colistin; AMC, Amoxicillin clavulanate; TZP, Piperacillin/Tazobactam; AMP, Ampicillin; CPM, Cefepime; CTX, Cefotaxime; FOX, Cefoxitin; CAZ, Ceftazidime; FOS, Fosfomycin.

Isolate ID	GEN	AMK	IPM	MEM	ETP	CIP	TMP/SMX	TGC	COL	AMC	TZP	AMP	CPM	CTX	FOX	CAZ	FOS	Carbapenemase Class (Enzyme)	MLST
**KpMO1**																		A	512
**KpMO2**																		A	512
**KpMO3**																		A	512
**KpMO4**																		A	512
**KpMO5**																		A	512
**KpMO6**																		A	512
**KpMO7**																		A	258
**KpMO8**																		A	512
**KpMO9**																		A	512
**KpMO10**																		A	512
**KpMO12**																		A	512
**KpMO14**																		A	512
**KpMO15**																		A	512
**KpMO16**																		A	512
**KpMO17**																		A	512
**KpMO19**																		A	512
**KpMO20**																		A	512
**KpMO21**																		A	512
**KpMO22**																		A	512
**KpMO23**																		A	512
**KpMO24**																		A	512
**KpMO25**																		A	512
**KpMO26**																		A	512
**KpMO27**																		A	512
**KpMO28**																		A	258
**KpMO29**																		A	512
**KpMO31**																		A	512

**Table 2:** PROVEAN analysis of new mutations in colistin-resistance related genes.

Gene	Nucleotide Mutation	Protein Variant	PROVEAN Score	Prediction (Cutoff = −2.5)
*mgrB*	Δnt61/70	T21L	−2996	Deleterious
		Q22T	−6000	Deleterious
		M23_W47del	−125,068	Deleterious
*phoQ*	C168A	S56R	−3.359	Deleterious
	ins799/801(GAC)	D266_K267insD	−8.067	Deleterious
	T260C	L87P	−4.65	Deleterious
*pmrB*	T137A	V46E	−4.063	Deleterious
	C284T	P95L	−9.604	Deleterious

**Table 3:** Isolates and patients’ characteristic. ^a^, BAL, Bronchoalveolar lavage; ^b^, Charlson Comorbidity Index; c, ICU, Intensive Care Unit; * Colistin treatment days before CRCR-*Kp* isolation (9 millions UI loading dose, then 4.5 millions twice a day).

Isolate ID	Sample Type ^a^	Collection Date	Age	Gender	CCI ^b^	Colistin Use* (Days)	Admission Ward ^c^ (Date)	2^nd^ Admission Ward (Date)	3^rd^ Admission Ward (Date)	Discharge/Death (Date)	Clinical Outcome
**KpMO1**	rectal swab	13/03/2013	82	M	3	no	ICU (25/02/2013)	Pneumology (01/03/2013)		Pneumology (27/03/2013)	recover
**KpMO2**	rectal swab	24/05/2013	68	F	3	no	Nephrology (23/05/2013)	−		Nephrology (06/06/2013)	chronic disease
**KpMO3**	rectal swab	24/07/2013	63	M	5	no	Nephrology (22/07/2013)			Nephrology (25/07/2013)	chronic disease
**KpMO4**	rectal swab	21/03/2013	77	M	2	yes (13)	Medicine II (28/02/2013)			Medicine II (23/03/2013)	death for sepsis
**KpMO5**	rectal swab	11/12/2013	78	F	3	no	Transplant Unit (08/11/2013)	ICU (01/12/2013)		ICU (17/12/2013)	death for non-infectious cause
**KpMO6**	rectal swab	02/12/2013	67	M	4	no	ICU (24/10/2013)	Infectious Disease (02/12/2013)	Long Term Care (26/02/2014)	Long Term Care (28/03/2014)	chronic disease
**KpMO7**	rectal swab	22/01/2013	71	F	2	no	Transplant Unit (22/01/2013)			Transplant Unit (05/03/2013)	recover
**KpMO8**	rectal swab	24/01/2013	68	M	6	no	Medicine-ICU (26/12/2012)	Nephrology (09/01/2013)		Nephrology (11/02/2013)	chronic disease
**KpMO9**	rectal swab	25/03/2013	73	F	2	no	Otolaryngology 05/02/2013	ICU (24/02/2013)	Pneumology (27/03/2013)	Pneumology (17/04/2013)	recover
**KpMO10**	urine	05/04/2013	57	M	3	no	Nephrology (23/03/2013)			Nephrology (22/05/2013)	recover
**KpMO12**	rectal swab	05/12/2013	70	F	8	no	Oncology (18/11/2013)	Pneumology (26/11/2013)		Pneumology (07/12/2013)	death for non-infectious cause
**KpMO14**	rectal swab	02/05/2013	55	F	4	yes (30)	ICU (20/02/2013)	Pneumology (02/03/2013)	Nephrology (19/03/2013)	Nephrology (11/06/2013)	chronic disease
**KpMO15**	rectal swab	09/01/2014	46	F	6	no	Infectious Disease (30/11/2013)			Infectious Disease (22/01/2014)	recover
**KpMO16**	blood	20/11/2013	73	M	3	no	ICU (21/10/2013)	Pneumology (13/11/2013)		Pneumology (01/01/2014)	death for sepsis
**KpMO17**	BAL	29/05/2013	47	M	3	yes (12)	Transplant Unit (14/03/2013)	ICU (17/05/2013)		ICU (14/06/2013)	death for sepsis
**KpMO19**	rectal swab	09/12/2013	66	M	1	yes (14)	ICU (25/11/2013)	Infectious Disease (10/01/2014)		Infectious Disease (30/01/2014)	recover
**KpMO20**	urine	18/07/2013	80	F	2	no	Infectious Disease (21/03/2013)	Long Term Care (08/07/2013)		Long Term Care (30/07/2013)	chronic disease
**KpMO21**	rectal swab	21/01/2013	43	M	2	yes (26)	ICU (03/01/2013)	Pneumology (29/01/2013)		Pneumology (07/02/2013)	death for non-infectious cause
**KpMO22**	rectal swab	17/05/2013	91	M	2	no	Infectious Disease (23/04/2013)			Infectious Disease (30/05/2013)	chronic disease
**KpMO23**	rectal swab	26/11/2013	76	F	2	no	Oncology (20/11/2013)			Oncology (04/12/2013)	chronic disease
**KpMO24**	rectal swab	10/12/2013	88	M	4	no	Medicine-ICU (17/11/2013)	Long Term Care (18/12/2013)	Medicine-ICU (03/01/2014)	Medicine-ICU (04/01/2014)	death for sepsis
**KpMO25**	rectal swab	30/12/2013	91	M	11	no	Orthopaedics (07/12/2013)	Medicine II (17/12/2013)		Medicine II (13/01/2014)	recover
**KpMO26**	rectal swab	22/05/2013	89	F	2	no	Nephrology (30/04/2013)			Nephrology (22/05/2013)	chronic disease
**KpMO27**	rectal swab	12/07/2013	94	F	4	no	Infectious Disease (22/06/2013)			Infectious Disease (14/07/2013)	death for sepsis
**KpMO28**	urine	07/03/2014	24	M	6	no	Infectious Disease (11/02/2014)			Infectious Disease (11/03/2014)	recover
**KpMO29**	rectal swab	16/12/2013	60	F	1	no	Medicine II (03/12/2013)			Medicine II (24/12/2013)	recover
**KpMO31**	rectal swab	09/12/2013	85	M	4	no	Medicine II (28/11/2013)			Medicine II (18/12/2013)	death for non-infectious cause

**Table 4:** Reference sequences used for BLAST analysis of chromosomal colistin resistance related genes. The *Kp*ST512-K30BO and *Kp*-HS11286 choice was due to their belonging to the same Clonal Complex of our isolates (CC258).

Head		Reference Sequences	GenBank Accession Number
Chromosomal loci	*acrAB*	*K. pneumoniae subsp. pneumoniae* ST512-K30BO (*Kp*-ST512-K30BO)	NZ_CAJM00000000.2
	*pmrHFIJKLM*		
	*crrAB*		
	*KpnEF*		
	*lpxM*	−	−
	*mgrB*	*K. pneumoniae subsp. pneumoniae* HS11286	CP003200.1
	*mgrB* promoter		
	*phoPQ*		
	*pmrABCD*		

## Supplementary Materials

The following are available online at https://www.mdpi.com/2079-6382/9/5/246/s1, Supplementary Table S1: MICs (μg/mL) of antimicrobial agents for CRCR-*Kp.* Supplementary Table S2: The assembly statistics for each genome.
