# Supplementary material for: A Retrospective Whole-Genome Sequencing Analysis of Carbapenem and Colistin-Resistant Klebsiella pneumoniae Nosocomial Strains Isolated during an MDR Surveillance Program
Source: Antibiotics (Basel). 2020 May 12;9(5):246. doi: 10.3390/antibiotics9050246 (PMC7277725; doi:10.3390/antibiotics9050246)
Supplement: Supplementary file 1 [file antibiotics-09-00246-s001.pdf]

Table S1. MICs (µg/mL) of antimicrobial agents for carbapenemresistant CRCR-*Kp.*

Isolate ID	GEN	AMK	IPM	MEM	ETP	CIP	TMP/SMX	TGC	COL	AMC	TZP	AMP	CPM	CTX	FOX	CAZ	FOS
KpMO1	4	≥64	≥16	≥16	≥8	≥4	≥320	4	≥16	≥32	≥128	≥32	≥64	≥64	≥64	≥64	64
KpMO2	4	≥64	≥16	≥16	≥8	≥4	≥320	4	≥16	≥32	≥128	≥32	≥64	≥64	≥64	≥64	64
KpMO3	4	≥64	≥16	≥16	≥8	≥4	≥320	4	4	≥32	≥128	≥32	≥64	≥64	≥64	≥64	128
KpMO4	4	≥64	≥16	≥16	≥8	≥4	≥320	≥8	≥16	≥32	≥128	≥32	≥64	≥64	≥64	≥64	128
KpMO5	4	≥64	≥16	≥16	≥8	≥4	≥320	2	4	≥32	≥128	≥32	≥64	≥64	≥64	≥64	64
KpMO6	4	≥64	≥16	≥16	≥8	≥4	≥320	2	≥16	≥32	≥128	≥32	≥64	≥64	ND	≥64	128
KpMO7	≤1	≥64	≥16	≥16	≥8	≥4	≥320	4	≥16	16	≥128	≥32	≥64	≥64	≥64	≥64	32
KpMO8	4	≥64	≥16	≥16	≥8	≥4	≥320	4	≥16	≥32	≥128	ND	≥64	≥64	≥64	≥64	64
KpMO9	4	≥64	≥16	≥16	≥8	≥4	≥320	4	≥16	≥32	≥128	≥32	≥64	≥64	≥64	≥64	128
KpMO10	4	≥64	≥16	≥16	≥8	≥4	≥320	≥8	≥16	≥32	≥128	≥32	≥64	≥64	≥64	≥64	64
KpMO12	8	≥64	≥16	≥16	≥8	≥4	≥320	4	≥16	≥32	≥128	≥32	≥64	≥64	≥64	≥64	≥256
KpMO14	≥16	≥64	≥16	≥16	≥8	≥4	≤20	≥8	≥16	≥32	≥128	≥32	≥64	≥64	≥64	≥64	≥256
KpMO15	2	≥64	≥16	≥16	≥8	≥4	≥320	2	≥16	≥32	≥128	≥32	8	8	32	≥64	≥256
KpMO16	4	≥64	≥16	≥16	≥8	≥4	≥320	2	8	≥32	≥128	≥32	≥64	≥64	≥64	≥64	128
KpMO17	4	≥64	≥16	≥16	≥8	≥4	≥320	4	≥16	≥32	≥128	≥32	≥64	≥64	≥64	≥64	64
KpMO19	4	≥64	≥16	≥16	≥8	≥4	≤20	2	≥16	≥32	≥128	≥32	≥64	≥64	≥64	≥64	64
KpMO20	4	≥64	≥16	≥16	≥8	≥4	≤20	4	≥16	≥32	≥128	≥32	≥64	≥64	≥64	≥64	64
KpMO21	4	≥64	≥16	≥16	≥8	≥4	≥320	4	≥16	≥32	≥128	≥32	≥64	≥64	≥64	≥64	64
KpMO22	4	≥64	≥16	≥16	≥8	≥4	≤20	4	≥16	≥32	≥128	≥32	≥64	≥64	≥64	≥64	128
KpMO23	4	≥64	≥16	≥16	≥8	≥4	≥320	≥8	≥16	≥32	≥128	≥32	≥64	≥64	≥64	≥64	128
KpMO24	4	≥64	≥16	≥16	≥8	≥4	≥320	2	8	≥32	≥128	≥32	≥64	≥64	≥64	≥64	128
KpMO25	≥16	≥64	≥16	≥16	≥8	≥4	≥320	2	4	≥32	≥128	≥32	≥64	≥64	≥64	≥64	≥256
KpMO26	4	≥64	≥16	≥16	≥8	≥4	≤20	4	≥16	≥32	≥128	≥32	≥64	≥64	≥64	≥64	128
KpMO27	4	≥64	≥16	≥16	≥8	≥4	≤20	4	≥16	≥32	≥128	≥32	≥64	≥64	≥64	≥64	128
KpMO28	≤1	≥64	≥32	≥16	≥8	≥4	≥320	4	≥16	≥32	≥128	≥32	16	≥64	≥64	≥64	64
KpMO29	4	≥64	≥16	≥16	≥8	≥4	≥320	2	≥16	≥32	≥128	≥32	≥64	≥64	≥64	≥64	128
KpMO31	4	≥64	≥16	≥16	≥8	≥4	≥320	2	≥16	≥32	≥128	≥32	≥64	≥64	≥64	≥64	64

GEN, Gentamicin; AMK, Amikacin; IPM, Imipenem; MEM, Meropenem; ETP, Ertapenem; CIP, Ciprofloxacin; TMP/SMX, Trimethoprim /Sulphonamide; TGC, Tigecycline; COL, Colistin; AMC, Amoxicillin clavulanate; TZP, Piperacillin/Tazobactam; AMP, Ampicillin; CPM, Cefepime; CTX, Cefotaxime; FOX, Cefoxitin; CAZ, Ceftazidime; FOS, Fosfomycin.

Table S2. The assembly statistics for each genome.

Sample	#reads	#trimmed reads	%	n contigs	n contigs >500	N50	Assembled Genome Size	Coverage	Assembler
KpMO1	2,097,234	1,974,712	0.94	436	226	77,317	5,828,912	166	Abyss
KpMO2	1,627,594	1,523,022	0.94	313	180	117,585	5,802,799	129	Abyss
KpMO3	1,599,364	1,481,416	0.93	346	208	93,645	5,900,018	123	Abyss
KpMO4	2,522,626	2,414,576	0.96	145	121	270,454	5,649,984	192	Spades
KpMO5	1,653,772	1,565,191	0.95	320	168	114,473	5,864,030	131	Abyss
KpMO6	853,184	822,321	0.96	142	117	231816	5,838,095	85	Spades
KpMO7	1,474,561	1,390,894	0.94	268	133	130,847	5,647,476	121	Abyss
KpMO8	1,519,782	1,426,441	0.94	360	220	73,691	5,799,329	121	Abyss
KpMO9	1,259,388	1,121,656	0.89	351	188	101,729	5,819,470	94	Abyss
KpMO10	866,690	758,185	0.87	405	217	72,732	5,796,677	64	Abyss
KpMO12	1,942,570	1,729,664	0.89	312	169	130,407	5,865,598	144	Abyss
KpMO14	1,117,562	1,002,531	0.90	301	142	117,479	5,884,692	83	Abyss
KpMO15	2,622,109	2,410,527	0.92	147	126	224,937	5,834,320	193	Spades
KpMO16	866,919	828,725	0.96	143	118	219807	5,838,580	85	Spades
KpMO17	2,635,452	2,421,013	0.92	149	134	209,780	5,839,796	194	Spades
KpMO19	778,548	744,094	0.96	99	84	219808	5,634,326	79	Spades
KpMO20	2,885,873	2,696,195	0.93	109	91	219,210	5,740,581	216	Spades
KpMO21	2,516,637	2,322,094	0.92	96	78	246,120	5,741,799	188	Spades
KpMO22	1,576,117	1,415,963	0.90	263	148	107,537	5,760,332	120	Abyss
KpMO23	778,667	739,154	0.95	152	128	203938	5,835,755	76	Spades
KpMO24	3,469,448	2,599,604	0.75	242	152	165,417	5,696,299	137	Spades
KpMO25	2,315,260	2,155,427	0.93	134	115	270,780	5,840,272	170	Spades
KpMO26	2,627,899	2,463,737	0.94	111	88	219,210	5,738,991	197	Spades
KpMO27	1,684,211	1,437,629	0.85	141	123	203,938	5,737,509	126	Spades
KpMO28	782,655	669,841	0.86	548	216	73,385	5,809,895	56	Abyss
KpMO29	850,985	575,868	0.68	355	246	73,635	5,788,323	30	Spades
KpMO31	1,633,959	1,562,692	0.96	133	112	270780	5,839,217	161	Spades
